# The Evolutionarily Mismatched Impact of Urbanization on Insomnia Symptoms: a Short Review of the Recent Literature

**DOI:** 10.1007/s11920-021-01239-7

**Published:** 2021-04-02

**Authors:** Jiaqing O, Catrin Pugh-Jones, Bethany Clark, Jenna Trott, Lei Chang

**Affiliations:** 1 Department of Psychology, Aberystwyth University, Aberystwyth, UK; 2 Plymouth, UK; 3 Department of Psychology, University of Macau, Macau, China

**Keywords:** Insomnia symptoms, Urbanization, Evolutionary mismatch, Review, Sleep, Modern environment

## Abstract

**Purpose of Review:**

For the most part of human existence, individuals have been living a rural lifestyle in a rural setting. However, such sleep-conducive conditions have largely been transformed dramatically by urbanization within a relatively short span of time in recent history, and the resulting evolved mechanisms-environment mismatch is theorized to bring about an increased risk for insomnia symptoms. This brief review of the recent literature is designed to evaluate the veracity of this proposition.

**Recent Findings:**

The majority of recent findings have suggested that most proposed evolutionarily mismatched urban factors are indeed related to the presence of insomnia symptoms. However, there is a general paucity of longitudinal evidence (and for some other factors, a lack of enough evidence of any kind).

**Summary:**

Although there is a preponderance of recent findings indicating a link between evolutionarily mismatched urban phenomena and insomnia symptoms, more longitudinal data are needed before any causative conclusion can be drawn.

## Introduction

*Homo sapiens* have spent the lion’s share of their existence residing in extremely rural environments. In fact, it has taken modern humans hundreds of thousands of years before around 30% of the global population were considered urban dwellers in 1950, although less than 7 decades were required for this proportion to exceed 55% [1, 2]. This evolutionarily very recent rapid rate of urbanization has likely created an evolutionary mismatch situation in that urban humans at present would not have the opportunity (e.g., the numerous generations of gradual evolutionary changes that are generally needed) to adapt adequately to a very different living environment, which could impact one’s well-being in a variety of ways. One of the most prominent effects could be in relation to sleep—an essential activity every human being would engage in for a substantial portion of their daily lives.

Indeed, a host of empirical evidence has suggested that urban inhabitants were more susceptible to developing insomnia symptoms than their rural counterparts (e.g., [3–5]). Through the lens of evolutionary mismatch, the evolved capacity to remain awake (or to be awakened) at night, for a certain duration at least, to attend to well-being issues is believed to be at a heightened risk of being overactivated in evolutionarily unfamiliar urban areas [6••]. While such an adaptation would conceivably have served an important survival function in the prehistoric context (e.g., keeping a lookout for a possible attack by an adversarial tribe), it could be erroneously triggered immoderately and unnecessarily by a range of evolutionarily different, usually much less hazardous, urban conditions [6••, 7].

### Differences Between Urban and Rural/Evolutionarily Familiar Conditions

For instance, because urban inhabitants are more likely to be residing alone than those in rural areas [8], they might be more sensitive to any potential signs of danger at night (e.g., be highly alert upon hearing some noises in the backyard) and are hence unable to sleep during such an occasion because they are conceivably concerned about having to deal with a possible problem (e.g., someone breaking into the house) on their own (even though it might just be noises generated by an intruding wild cat). Such concerns and sleepless experiences are believed to have been much less prevalent and pronounced among virtually all ancient human societies (as data from extant hunter-gatherers, generally regarded as a reasonable representation of them, would suggest) and even those that had existed more recently on the evolutionary timeline prior to modern developments, despite the greater abundance of threats, because practically everyone was living in close proximity with other individuals [9, 10]. Research suggested that this evolutionarily almost ubiquitous residential way of life would have afforded occupants a greater sense of security (and therefore a much lower risk of sleep difficulties) especially due to evolved differences in age-dependent sleep-wake timings among different members of the household such that at least one person was nearly always alert [11•].

Empirical evidence has also suggested that these prehistoric groups were broadly made up of biological and affinal relatives [10]—individuals who would have been protective of each other’s well-being in general due to it being an important determinant of the evolutionary success of their (future) biologically related descendants [12••]. This is in stark contrast to the relatively common evolutionarily recent phenomenon of solo-living, or living apart from and having relatively irregular interactions with most of one’s kin, especially among urban-dwelling individuals [8, 9, 13]. Thus, in light of the empirically suggested stress-alleviating role of kin support [14, 15] and the knowledge that sleep difficulties can result from the pressures of life [16], it is believed that the comparative lack of easily accessible support from relatives, in addition to the issues surrounding solo-living itself, is likely a risk factor for developing insomnia symptoms among urban residents [17].

This heightened vulnerability to developing insomnia symptoms might be further exacerbated by other evolutionarily unnatural urban lifestyle behaviors. More specifically, the relative lack of engagement in physical activity among urban inhabitants (as research would suggest) [18], coupled with their general tendency to spend more time in front of a screen [19, 20], could presumably contribute to more sleep difficulties [21, 22]. Long before the advent of blue light-producing (and hence melatonin-reducing) electronics [23], ancestral humans were believed to have been very physically active (e.g., typically covering a distance spanning numerous kilometers) in their daily lives [24••]. Modern urban environments have provided an evolutionarily unprecedented array of technological conveniences that largely negates the need for much physical activity, but our evolved psychobiological mechanisms are conceivably designed for an active way of living and such a mismatch will potentially result in more (mental) health issues (e.g., in the form of insomnia complaints).

An urban lifestyle and residential environment might, nevertheless, not be the only evolutionarily mismatched factors that could play a role in enhancing one’s risk of experiencing insomnia symptoms. The conditions surrounding one’s home are also important considerations. For example, not only are urban dwellers more likely to be living alone and/or have less support from their kin (as discussed above), they are also more likely to inhabit a densely populated community full of people they are unacquainted with (while being surrounded by sparse greenery). This relative lack of affiliation and a sense of togetherness with those living in one’s community [25] could likewise induce a state of psychological tension which is predictably detrimental to sleep health [16], as could the comparative inadequacy of contact with nature among these urban residents [26]. These urban idiosyncrasies are theorized to be stress-inducing because they are an indication that the surroundings are bereft of the types of peaceful conditions conducive for thinking and working through stressors (e.g., a relative absence of unacquainted individuals—who are believed to be more likely to jeopardize one’s welfare throughout prehistory—and the presence of certain reflection-friendly unthreatening natural spaces) which humans have evolved to be adapted to [7].

Other potential challenges to a good night’s sleep might also emerge due to changes arising from urbanization that has occurred in the vicinity of one’s dwelling. One of the most prominent of these predictable urban problems is the amount of light pollution from the widespread (and perhaps excessive) use of artificial lights at night. Besides urban inhabitants’ greater usage of blue light-producing electronics (as discussed above), they could also be much more exposed to an immoderate quantity of melatonin-reducing light in their immediate environment (e.g., from streetlamps and buildings) during nighttime than their rural counterparts (and certainly in comparison to their electricity-free, fire-utilizing prehistoric predecessors) [27].

This conceivable risk factor for the development of insomnia symptoms might even occur in conjunction with other forms of potentially sleep-disrupting pollution in one’s urban neighborhood at night. Indeed, empirical data have indicated that noise levels in urban areas were, as a whole, considerably greater compared to those in rural environments, mainly due to the presence of a larger number of moving vehicles [28]. In addition to the relatively greater number of vehicles on the road, excessive amount of nocturnal noise might expectedly also be produced in urban cities by individuals who could be out on the streets at night, typically in response to the wide variety of businesses (e.g., restaurants and pubs) that do operate until late night. Similar urban-rural differences regarding air pollution levels have also been observed [29], which were likely due to emissions from the higher number of vehicles and factories in urban areas.

The noises and odors generated by these pollution sources are likely to activate one’s evolved mechanisms associated with threat detection, prompting one to become alert and therefore less able to fall asleep (or to return to sleep). This is because the presence of any nocturnal noise or unpleasant smell in the prehistoric context was often a sign of immediate danger (e.g., a carnivore was in the vicinity or there was an unintended fire), and hence, it would have been beneficial for one to be vigilant in such situations (although there were usually other residents who would be awake at any one time to ensure everything was fine or to wake everyone up if needed, as indicated above). Due to evolutionary mismatch, however, comparatively safer (albeit still directly/indirectly harmful to well-being if chronically exposed) disturbances resulting from these forms of pollution could yet induce one to have sleepless experiences during bedtime on a regular basis in the modern urban context.

Taken together, considering all these proposed problems of urbanization, it would seem reasonable to surmise that insomnia symptoms are largely an unfortunate byproduct of living in an evolutionarily unfamiliar environment. Nevertheless, while plenty of studies have indeed suggested that urban surroundings are more likely to facilitate the development of such experiences (as highlighted above) [3–5], some other research have pointed to the contrary (e.g., [30]) or have found no regional distinction in this regard (e.g., [31]). This is, in fact, not totally surprising though because an evolutionarily mismatch environment (e.g., an urban city) could nonetheless also bring about certain positive evolutionarily novel changes (e.g., the widespread ease of possessing modern inventions such as contemporary beds and heaters/air conditioners) which would predictably aid a better night’s sleep. The eventual nature of one’s sleep experiences would then hinge on whether the enhanced sense of comfort these modern possessions might afford would normally negate the conceivably more influential (if present) problems of urbanization. To this end, this article will assess if the recent literature does support the notion that these diverse micro and relatively more macro urban phenomena are indeed related to a heightened risk of developing insomnia symptoms via a brief review.

### Approach of This Brief Review of the Recent Literature

The aim of this brief review is to provide a very short, condensed overview of selected recent peer-reviewed research work (e.g., published in 2015 or later; reviews are not discussed because they would likely have covered older empirical evidence) that has investigated the role of at least one of the abovementioned evolutionarily relevant urban-prevalent factors on the occurrence of insomnia symptoms. It is not the intention of the review though to offer an exhaustive coverage of all journal articles that have been published on this topic to date. While several recent reviews have either concentrated on how certain environmental factors would play a role in the emergence of sleep problems/conditions more generally (with a limited focus on insomnia symptoms, if any) (e.g., [32, 33, 34••, 35–38]) or how methodological and demographical disparities (including the rural-urban contrast) might predict reports of inferior sleep health in relatively less well-to-do nations (e.g., [39•]) respectively, the current review is unique in its emphasis on evaluating the overall impact of a variety of urban-prevalent factors (regardless of geographical location) purely on the occurrence of insomnia symptoms as predicted by the perspective of evolutionary mismatch.

## Evolutionarily Mismatched Urban Phenomena and Insomnia Symptoms

### A Greater Tendency to Live Alone

A sizeable quantity of studies, conducted with an assortment of different sample groups, has investigated the influence of solo-living on the development of insomnia symptoms within the last few years, with the majority of them demonstrating the existence of a link between the two (e.g., [40–44]), although a few has bucked the trend (e.g., [45, 46]). For instance, solo-living was shown to be related to greater odds of heightened insomnia symptoms among various individuals including the elderly (e.g., with an odds ratio of “1.49”) (p. E94) [40], and type 2 diabetes sufferers (with an odds ratio of “2.22”) (p. 1877) [41]. However, the general absence of a robust array of longitudinal evidence that was obtained in recent years focusing specifically on the predictive value of solo-living has meant that any predictions regarding cause-and-effect remain generally speculative.

### Reduced Support from One’s Kin

Analogous to the existing pool of research on solo-living, there is also a preponderance of varied findings on the inverse relationship linking kin support with insomnia symptoms in the recent literature (e.g., [47–51]). For instance, a study on a group of Taiwanese youth found that having an inadequate amount of support from one’s kin was directly related to heightened insomnia complaints (“β = −0.06”) (p. 1) [47], while another study on a group of American teenagers has demonstrated that such an experience could also be indirectly predictive of greater insomnia complaints (in this case, at the conclusion of their initial high school year by means of its effect on such sleep difficulties at the start of it) (*B* = “−.01”) (p. 1033) [48]. This is in juxtaposition to a handful of other findings that have either contradicted these outcomes (e.g., [52]) or has suggested that the insomnia-mitigating benefit of help and comfort provided by others may not be exclusive to the support one can obtained from kin (e.g., [53]). Although there were emerging evidence demonstrating the existence of the kin support-insomnia symptoms relationship across a longer timeframe (e.g., [48]), most of the empirical investigations on this association were likewise carried out at a single time-point.

### A Less Active Lifestyle

The evidence base supporting the buffering role of physical activity on the development of insomnia symptoms appears to be comparatively more robust. Even though the current focus is just on the range of studies that were conducted since 2015, in contrast to the relatively small amount of contradictory findings (e.g., [54•]), there was already an overwhelming body of evidence suggesting that the adoption of an active lifestyle was linked to a curtailment in one’s probability of developing insomnia symptoms (e.g., [55, 56, 57•, 58–63]). More specifically, a good number of these research outcomes was also observed longitudinally (e.g., [56, 58, 59, 61]). For instance, Chen and colleagues reported that physically idle individuals were more likely, in juxtaposition to those who have been involved in physical pursuits, to develop insomnia issues (with a hazard ratio of “1.22”) across a span of 7 years (p. 189) [59], while similar outcomes were shown in a separate empirical investigation on female adults who have transformed from being physically idle to individuals who were intensely engaged in physical endeavors (versus those who have remained physically idle) (with an odds ratio of “0.17” after accounting for relevant factors) in a prospective investigation that spanned 10 years (p. 22) [58]. Such findings have provided a certain level of credibility to the proposed association when assessed in conjunction with the presence of accumulative data that was experimentally obtained over a period of time (e.g., [55, 60, 63]).

### (More) Screen Time

There is similarly a huge quantity of evidence endorsing the link between screen time, or the amount of time devoted to looking at digital content for work or leisure purposes via one or more of the diverse kinds of electronic equipment that are available in the modern world, and insomnia symptoms (e.g., [64–68]). These have encompassed evidence that have focused on activities that varied from television consumption (with an odds ratio of “2.49” and “1.72” for male and female youths respectively) (p. 700) [65] to electronic gaming (“β = 0.120”) (p. 189) [67] and their relationships with insomnia complaints. This has compared very favorably with the relatively much fewer number of contrary findings in the recent literature (e.g., [69•]). However, together with the presence of some mixed data focusing on how one’s dependency on a media platform/device could affect insomnia symptoms over a period of time (e.g., [70, 71]), a much greater array of pertinent longitudinal research work is needed to ascertain the validity of the specific causal relationship between screen time and insomnia symptoms.

### Less Closely Knit Communities

In contrast to the diversity of findings in relation to the influence of physical activity (and to a lesser extent, screen time), there were relatively fewer pieces of empirical evidence on how one’s sense of connection with their neighbors/community and how dependable they are could affect the presence of insomnia symptoms. On the whole, there is a broad trend suggesting that such a sense of connectedness/belief was linked to lesser insomnia symptoms, with most studies advocating this general view (e.g., [40, 72–74]). As an example, findings from Alhasan and colleagues’ study have suggested that insomnia complaints were more pronounced among individuals residing in an area with little “neighborhood social cohesion” in contrast to their more closely knit counterparts (with a prevalence ratio of “1.26” after accounting for other relevant factors) (p. 7) [73]. In a similar vein, Sugawara and colleagues reported that having the perception that those in one’s community were undependable months following a natural disaster was predictive of greater insomnia issues over the subsequent 6-year span (*B* ranges from “−0.007” to “−0.013” in different analytical models that have controlled for different relevant factors) (p. 5) [74]. Yet, like some of the evidence base associated with the other factors, the overall dearth of a healthy assortment of longitudinal findings (or even just enough relevant findings in general), with the exception of some initial data (e.g., [74]), has rendered it difficult to make any clear-cut conclusion.

### Lesser Exposure to Natural Spaces

In a fairly similar fashion to the findings regarding the role of one’s connectedness to/trust in one’s community, there were not many studies that have explored the relationship between nature exposure and insomnia symptoms in recent years (and the available evidence have appeared mixed). For instance, results from a study that was carried out on a psychiatric sample seemed to back the insomnia symptoms-mitigating idea of nature exposure (although they did not provide relevant specific figures for the findings) [75], while results from another study have painted a more mixed picture whereby walking in an older forest (but not in a forest with newer trees) over a number of days was shown to lead to a reduction of the frequency of insomnia complaints (with a difference score of “−4.7” days although the *Z* statistic was not reported for the Wilcoxon signed-ranks test) (p. 6) [76]. In addition, results from another separate study, for example, have instead not revealed any benefit from a “forest therapy” (which spanned a number of days) on insomnia complaints (pre-test insomnia issues scores = “12 ± 5.2” versus post-test insomnia issues scores = “11 ± 5.5”) (p. 5) [77]. Furthermore, methodological issues with some of these research work have likely muddied the waters further, such as the possibility that part of the outcomes might have occurred primarily due to the theoretical unsuitability of one of the stimuli [76], or that it is challenging to tease apart the individual impact of each factor in a mixture of stimuli that might differ in terms of effectiveness in addressing insomnia symptoms as they were mainly presented together to the participants as a whole [75, 77]. Much more updated research work is needed to cast more light on the validity of the argument that a lack of contact with nature is a determining factor in the emergence of insomnia symptoms.

### Greater Exposure to Artificial Light at Night

The overall picture regarding the relationship between individuals’ exposure to artificial light during nighttime and their insomnia symptoms risk, on top of potential issues with the use of blue light-emitting electronic equipment as reviewed earlier, is mainly an uncertain one as well. While there were studies suggesting that such a link is accurate (e.g., [78, 79]), others have produced contrary findings (e.g., [80, 81]). For instance, findings like those from Min and Min have revealed that greater exposure to human-made light stemming from outside of one’s residence was related to more severe insomnia complaints (operationalized as greater prescribed quantity of sleeping pills [e.g., *B* = “18.20” in terms of all sleeping pills for those who were most exposed after accounting for relevant factors] and more prolonged consumption of these medications [e.g., *B* = “14.03” in terms of all sleeping pills for the most exposed after accounting for relevant factors]) (p. 1907) [78]; these outcomes, however, contrasted with other findings such as those from Desaulniers and colleagues which showed that a more illuminated bedroom during nighttime was not related to greater odds (odds ratio of “0.88”) of experiencing heightened insomnia complaints (p. 4) [80]. Coupled with the fairly limited quantity of studies assessing this phenomenon, these mixed findings suggest that the jury is still out on the predictive value of light pollution on the development of insomnia symptoms based on recent evidence alone.

### Higher Level of Noise Pollution from Outdoors

In the main, there is a relatively huge predominance of recent outcomes substantiating the assertion that noise disturbances in the vicinity of one’s residence is linked to increased insomnia symptoms (e.g., [69•, 82–84]). These include the relationships auditory disturbances generated by aircraft in the vicinity (with an odds ratio that was greater than 3 across different analytical models and groups that varied in terms of the severity of such form of pollution) [84] and by surrounding traffic have with insomnia complaints (with an odds ratio that varied from “2.95” to “3.54” for different types of insomnia complaints) (p. 545) [82]. This has greatly contrasted with the much smaller minority of evidence that has either challenged this overall impression (e.g., [85]) or has afforded a more ambivalent view of this issue (e.g., [86]). A general lack of a wide range of solid longitudinal data has prevented a more definite causal interpretation of this plausible connection between noise pollution from outdoors and one’s susceptibility to develop insomnia symptoms.

### Poorer Air Quality from Outdoors

There were very few studies that have specifically evaluated the influence of air pollution in one’s residential surroundings on insomnia symptoms risk in the last few years. The hugely limited selection of recent evidence has generally suggested that such an association does exist (e.g., [82, 87, 88]). Some of these findings were not entirely clear-cut though. For instance, although findings from a study by Purnama and colleagues have appeared to suggest that poorer air quality due to the atmospheric release of lead by vehicles (among other threats) was associated with greater insomnia complaints, such outcomes could conceivably have been confounded by other relevant variables that were not controlled for in the study [88]. In a similar fashion, while findings from a separate study by Janson and colleagues have analogously shown that poorer air quality (operationalized as one’s closeness to surrounding traffic in the absence of related auditory disturbances) was linked to a major type of insomnia complaints (with an odds ratio of “1.62”), it was not found to be related to other types of insomnia symptoms (p. 545) [82]. A much larger variety of research work is required before any reasonable causative conclusion can be drawn regarding the role of air pollution from outdoors on insomnia symptoms.

### More Potentially Sleep-Benefitting Modern Bedroom Items

In a somewhat similar vein to research work on air pollution, there was not a lot of evidence that was obtained recently on the relationship between possessing sleep-enhancing urban-prevalent bedroom items (e.g., a [higher-quality] bed, pillow, heater, dehumidifier and/or air conditioning unit) or the presence of indicators implying a lack of these items (e.g., an uncomfortably warm/cold/damp sleeping area) and the occurrence of insomnia symptoms. The handful of available direct/indirect findings has either yielded mixed outcomes (e.g., [69, 80]), suggested that such a proposed link is justifiable in general (e.g., [89]), or has questioned its authenticity (e.g., [90•]).

More specifically, while Desaulniers and colleagues have reported that having a pillow that one was not/fairly contented with (as opposed to a pillow that one was highly contented with—an item that is likely to be more prevalent in the modern urban environment) was related to higher odds (odds ratio = “2.00”) of insomnia complaints, such a link was not demonstrated for an unpleasant/fairly pleasant mattress (versus a highly pleasant one—another item that is arguably and similarly more prevalent in the urban context) (odds ratio = “0.97”) (p. 4) [80]. These outcomes contrasted somewhat with other findings, such as those from a study by Wang and colleagues which demonstrated that the existence of certain conceivable signs of humidity inside one’s house (e.g., the presence of mold or a slightly wet floor [but not the smell of mold however]), arguably due to the lack of more urban-prevalent items like a dehumidifier, was related to greater odds (with odds ratios between “1.30” to “1.70”) of insomnia complaints when assessed after a 10-year period (p. 5) [89]; and findings such as those from Jansson-Fröjmark and colleagues which demonstrated that factors like having an unpleasant bed or an unpleasantly cold/hot bedroom (which could conceivably be due to the lack of more urban-prevalent items like a heater or an air-conditioning unit) were not predictive of greater insomnia complaints (the actual statistical figures were not reported in the study) [90•]. Therefore, a much more robust collection of updated empirical results will analogously be needed to determine the veracity of this hypothesis.

## Conclusion

Taken together, this brief review of the recent literature has demonstrated that findings were largely in line with the theoretical assumption that urbanization could influence insomnia symptoms risk. Nevertheless, with the exception of research work pertaining to the adoption of an active lifestyle, a general shortage of longitudinal data has precluded any conclusive interpretation of the relationship between any of the proposed urban-prevalent factors and the development of insomnia symptoms. There were likewise too few studies of any kind where factors such as community cohesion/trust and air quality are concerned, whereas outcomes were additionally mixed in general for other determinants including nature exposure, light pollution, and the presence of modern bedroom items.

Thus, in order to ascertain if evolutionarily mismatched urban phenomena are indeed important causal contributors to insomnia symptoms risk in the urban world, several important research endeavors should be adopted in the future. In particular, there should be (1) more empirical investigations on the role of the abovementioned factors on insomnia symptoms, specifically those that are conducted over a longer period of time and/or experimentally (where ethically appropriate for some of the identified factors); (2) empirical examinations evaluating the predictive value of the evolutionary mismatch argument in juxtaposition to other traditional theories of insomnia symptoms; and (3) a more comprehensive review (e.g., a systematic review that covers an exhaustive range of relevant articles over a much longer timeframe) to provide a clearer picture of these hypothesized phenomena.
